# Predicting Drug Resistance Using Deep Mutational Scanning

**DOI:** 10.3390/molecules25092265

**Published:** 2020-05-11

**Authors:** Gur Pines, Reilly G. Fankhauser, Carrie A. Eckert

**Affiliations:** 1 Department of Entomology, Agricultural Research Organization, Volcani Center, P.O.B 15159, Rishon LeZion 7505101, Israel; 2 Department of Dermatology, Oregon Health & Science University, Baird Hall 3225 SW Pavilion Loop, Portland, OR 97239, USA; fankhaur@ohsu.edu; 3 Renewable and Sustainable Energy Institute, University of Colorado Boulder, 027 UCB, Boulder, CO 80309, USA; 4 Biosciences Center, National Renewable Energy Laboratory, 15013 Denver West Parkway, Golden, CO 80401, USA

**Keywords:** sequence to activity mapping, genome editing, fosmidomycin, DXR, drug resistance

## Abstract

Drug resistance is a major healthcare challenge, resulting in a continuous need to develop new inhibitors. The development of these inhibitors requires an understanding of the mechanisms of resistance for a critical mass of occurrences. Recent genome editing technologies based on high-throughput DNA synthesis and sequencing may help to predict mutations resulting in resistance by testing large mutagenesis libraries. Here we describe the rationale of this approach, with examples and relevance to drug development and resistance in malaria.

## 1. Introduction

In 1946, Alexander Fleming stated: “There is probably no chemotherapeutic drug to which in suitable circumstances the bacteria cannot react by in some way acquiring ‘fastness’ [resistance]” [1]. Today, resistance to drugs is considered unavoidable as multi-drug resistant infections become a serious problem and possibly mark the post-antibiotic age [2,3]. This inevitable resistance leads to an arms race where new-generation drugs are being developed continuously, only to be rendered useless upon the development of resistance in the target system.

**Figure 1:**
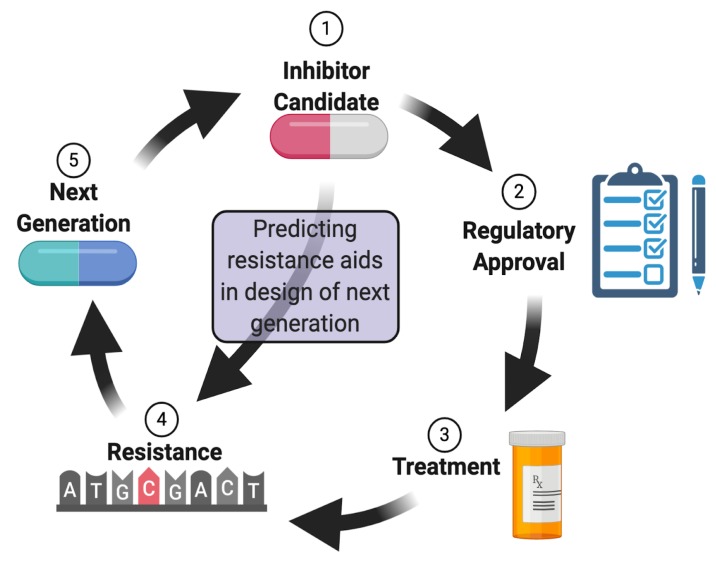
An illustration of the drug development cycle, focusing on mutationally acquired resistance. (1) A compound candidate is identified, and following rigorous testing, it is approved for clinical use (2). Patients are treated with the drug (3); and ultimately, resistance emerges, which is sometimes acquired via mutations in the target gene (4). As the drug becomes less effective, a new drug or a derivative of the current one enters the development pipeline (5). The prediction of resistant mutants before they occur in the field may increase the rate of development of new derivatives. (Illustration created with BioRender.com,).

This drug development cycle takes a tremendous amount of time and funds, as the resistance mechanism must be deciphered from initial anecdotal occurrences that appear spontaneously in the field (Figure 1). After the discovery of the exact resistance mechanism, new compounds or molecular derivatives of the original drugs must be tested for effectiveness both towards the wild type and resistant mutant. Finally, the selected inhibitor must undergo regulatory scrutiny until it is ultimately approved. As the trivial solutions are exhausted, every successive cycle is harder and more expensive than the preceding cycles, requiring the new drug to be effective against the wild type and each resistant form.

To accelerate this cycle, new and higher-throughput methods for discovery must be employed. One possible solution is to predict the resistance-conferring mutations by actively mutating the target gene and selecting for resistant mutants through directed evolution. Naturally, the more comprehensive and systematic the mutagenesis is, the higher the chances of achieving a complete prediction.

Here, we discuss various approaches for performing deep mutational scans and address the benefits and limitations of each strategy. We go on to give examples of how these tools have been successfully used to predict mutationally acquired resistance mechanisms in cases relevant to malaria. Lastly, we discuss how such technologies may further advance the understanding of resistance and identify drug–target pairs.

## 2. Directed Evolution

**Figure 2:**
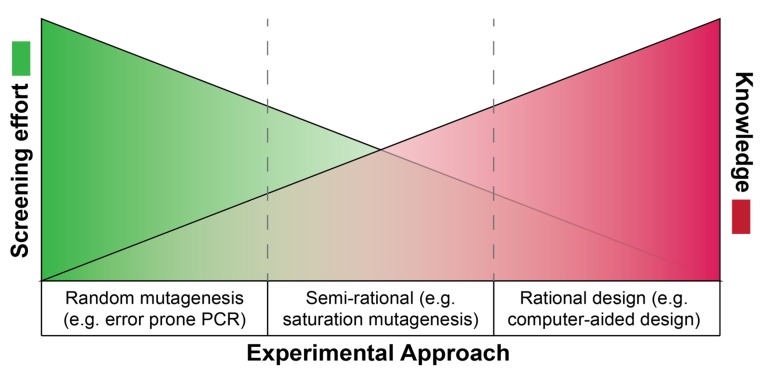
A knowledge vs. screening effort schematic. The more information accumulated about a specific protein, the more focused and rational the mutagenesis libraries can be, reducing the screening burden. Contrarily, when mutating a protein with no prior relevant knowledge, random methods will be employed, increasing the screening load.

Directed evolution experiments mimic the principles of natural selection at laboratory timescales. The induction of genetic diversity is followed by screening or selection steps to deplete the unfit mutants while preserving the more fit mutants that will be the target for the next round of mutagenesis [4]. Genomic diversity may occur naturally [5] or can be induced randomly using radiation, mutagenic chemicals, or engineered mutator strains [6,7,8,9]. More focused random mutagenesis methods include error-prone PCR and DNA shuffling (Figure 2). For error-prone PCR, the reaction is performed under non-ideal conditions or by using mutated polymerases to reduce overall fidelity and increase the mutation rate [10]. In DNA shuffling, multiple variants of a gene are used to create chimeras with modified properties [11]. On the other end of the spectrum are rational design approaches where structure-based computer-aided designs focus on a particular set of mutations that are predicted to achieve the desired trait [12,13,14]. Between random mutagenesis and rational design lie semi-rational approaches such as saturation mutagenesis. This method is employed when specific sites are a target, but the final identity of these sites cannot be predicted, and all, or a subset, of amino acids are converted to each other possible amino acid at that position [15,16]. Several approaches to controlling the size of saturated libraries have been reported [17,18,19], aiming to reduce downstream screening efforts.

The exact method for introducing this genetic diversity generally depends on two factors: (1) the available throughput for mutant testing and (2) the amount of knowledge available for the target gene. In the investigation of resistance, however, the throughput is mostly irrelevant since selection can be used for any library size, leaving the knowledge level as the primary consideration. Genes with no prior literature will be subjected to random mutagenesis approaches. In contrast, genes that have been studied extensively will be mutated in specific target regions. Targets for rational design may be derived from three-dimensional structures, computational sequence analysis, and naturally occurring mutations in homologous genes or domains, among others. Massively parallel DNA synthesis coupled with next-generation sequencing can support the systematic sequence-to-activity mapping by saturating every amino acid in a gene, generating a library of complete single amino acid polymorphisms. Following the selection step, where the library is treated with the drug of interest, the surviving population will harbor mutations that confer resistance—some of which may be unintuitive. Hence, the level of library completeness correlates with the confidence of isolating the complete repertoire of the resistance-conferring mutants.

## 3. Library Design

**Figure 3:**
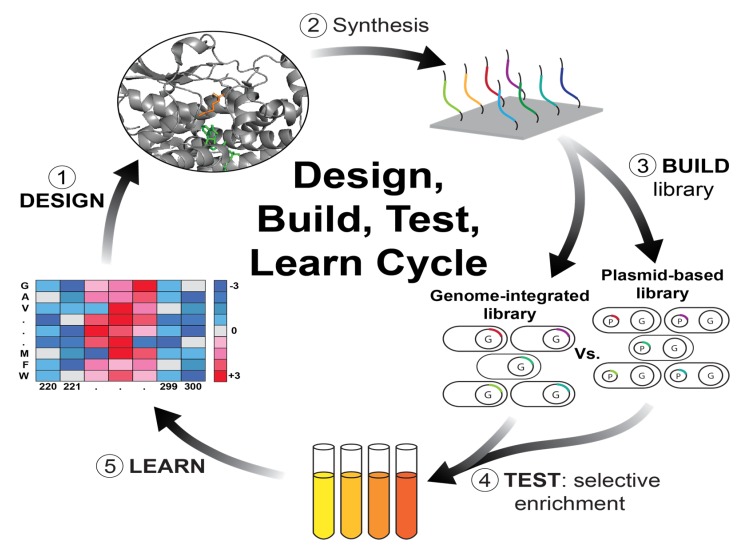
The Design–Build–Test–Learn cycle in the drug resistance study context. (1) Library design may be exhaustive, including all possible single amino acid mutations, or more focused on a specific domain of the target protein. The libraries are constructed, usually by using massively parallel DNA synthesis. (3) The library may be expressed on plasmids (P) or integrated into the genome (G). (4) Library cells are subjected to selection using the inhibitor of interest. (5) The target genes in the surviving cells are sequenced to infer the resistant mutants. These mutants may serve as templates for subsequent rounds if a multi-mutant phenotype is desired.

Strategies using deep scanning saturation mutagenesis libraries employ the “Design–Build–Test–Learn” cycle adopted from computational and engineering sciences (Figure 3) [20], and have been applied for several genes, spanning antibiotic-resistance genes in bacteria to oncogenes in human cells [21,22,23,24,25,26]. The complete mutational landscape of the beta-lactamase gene at single amino acid resolution was made by Firnberg [27] and Stiffler [28], providing a high-resolution picture of mutational fitness and drug resistance.

These libraries were made on plasmids, allowing the study of genes that are not necessarily expressed naturally in the subject organism. Assuredly, it is simpler to construct a library of variants and express them on a plasmid than to integrate each mutant onto the genome. However, lack of native regulation and variation in plasmid copy numbers leading to stochastic distributions of protein products can, in turn, affect fitness estimates [29,30,31,32]. Direct genome editing, when applicable, handles these concerns, but large-scale editing was, until recently, technologically infeasible. One possible approach for integrating mutants onto the genome involves error-prone PCR to generate a diverse library of mutants coupled to a CRISPR/Cas9 system to select for recombinants [33,34]. These approaches benefit from being relatively straightforward and inexpensive since they do not require large-scale DNA synthesis and rely on a single, pre-validated guide RNA. While these approaches ultimately rely upon random errors to generate the diversity of the library members, sufficiently large samples may statistically cover most single nucleotide substitutions. For applications where complete coverage of an entire gene or specific target regions is needed, a strategy utilizing synthetic libraries may be necessary. Examples of such approaches involving the genomic incorporation of synthetic libraries utilizing CRISPR/Cas9 systems as a selection tool are CRISPR-Enabled Trackable Genome Engineering (CREATE) and HI-CRISPR, among others [35,36,37,38,39]. Using the CREATE technology, we were able to search for point mutations in the *ispC/dxr* gene that confer resistance to the antimalarial fosmidomycin (3-(*N*-formyl-*N*-hydroxyamino)propylphosphonic acid (FSM), uncovering mutations not elucidated in previous error-prone PCR strategies (see below) [40,41].

An important consideration for library design is whether to perform complete or restricted saturation. In some cases, the best performing mutants, also termed the highest peaks of the mutational landscape [42], require a significant change in the chemical properties of the target amino acid, thus requiring a change in two or three bases in the codon [43,44,45]. While complete saturation mutagenesis, allowing access to the entire amino acid repertoire, may be necessary for basic studies and protein engineering efforts, for the study of naturally-occurring resistance phenotypes, it may only be required to restrict the library to single base changes, thus reducing downstream labor and cost. Another approach to reducing library size is to rationally select the targets for mutagenesis rather than systematically mutating the complete gene [40]. Such rationally designed libraries are only possible when a large enough body of knowledge is present, as described above. While this approach risks missing important mutations, it may balance between comprehensiveness and manageability.

While it has been shown that in some cases a single mutation might be enough [46], double and more mutants conferring increased resistance to various inhibitors have been reported [47,48,49]. Ideally, this space should be explored; however, a complete double mutation library (or more) is significantly more extensive and might prove unfeasible with current technologies. Another approach is to accumulate mutations iteratively by cycling through mutagenesis and selection to climb up the mutational landscape rather than testing all possible mutations [50,51].

## 4. The Example of Deoxyxylulose Phosphate Reductoisomerase

**Figure 4:**
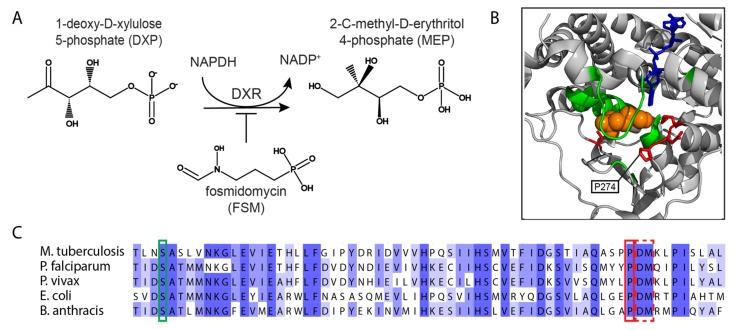
DXR is a target of fosmidomycin. (**A**) The reaction is catalyzed by DXR and inhibited by FSM. (**B**) The three-dimensional structure of DXR. The DXR structure is shown in gray (PDB#1Q0l), FSM is colored orange, and NADPH is in blue. Green amino acids represent the amino acids selected for mutagenesis, and mutations enriched following FSM incubation are colored red. This panel is adapted with permission from Pines et al. Genomic Deoxyxylulose Phosphate Reductoisomerase (DXR) Mutations Conferring Resistance to the malarial Drug Fosmidomycin in *E. coli*. ACS Synthetic Biology. Copyright 2018 American Chemical Society [40]. (**C**) Sites responsible for FSM resistance are highly conserved. The highlighted serine (green box) was identified by Armstrong et al. utilizing error-prone PCR. The proline (solid red box), and to a lesser extent, the adjacent aspartate and methionine residues (dashed red box) were found to confer FSM resistance using direct genome editing by Pines et al. More remote sites conferring resistance are not shown.

Deoxyxylulose phosphate reductoisomerase (DXR) is a key early enzyme in the non-mevalonate pathway, which catalyzes both the intramolecular rearrangement and reduction of 1-deoxy-d-xylulose 5-phosphate (DXP) to 2-*C*-methyl-d-erythritol 4-phosphate (MEP) (Figure 4A). The mevalonate pathway was long considered the exclusive pathway that produces isoprenoids such as steroid hormones, carotenoids, and ubiquinone or menaquinone. However, studies in the late 1980s and early 1990s suggested that an alternative non-mevalonate-related pathway exists—mainly in bacteria, plant plastids, and in a plastid-like organelle in *Plasmodium falciparum*, as well as other protozoan parasites of the phylum Apicomplexa [52]. Since most eukaryotes, including humans, use the mevalonate pathway, the non-mevalonate pathway is an appealing target for inhibition [53].

FSM was first isolated from *Streptomyces lavendulae* in 1980 [54,55] as a new antibacterial and was shown to inhibit isoprenoid biosynthesis in 1989 [56]. FSM showed an antibacterial spectrum that was consistent with the non-mevalonate pathway, leading to the isolation of its target, DXR [57]. Ten years later, FSM was considered as a potential antimalarial as the MEP pathway is highly conserved in *Plasmodium* species [58,59,60].

While FSM is effective in malaria, previous studies have demonstrated *P. falciparum* gaining resistance to FSM through changes in metabolic flux via the MEP pathway and amplification of the DXR gene [61,62]. Contrary to *P. falciparum,* both *Mycobacterium tuberculosis* and *Toxoplasma gondii* are natively resistant to FSM due to a lack of cellular drug intake [63,64]. DXR is a highly conserved enzyme in the non-mevalonate pathway, and FSM is effective to some extent in *E. coli*, with several reports of resistance related to lack of intake [65] and FSM export from the cell [66]. A DXR point mutation conferring resistance was identified via error-prone PCR in *E. coli* [41]. In addition, several *P. falciparum dxr* mutations were correlated with increased half-maximal inhibitory concentration (IC_50_) of FSM; however, further studies are required to determine causality [67]. As high-throughput tools for engineering *P. falciparum* have yet to be demonstrated, we took advantage of the conserved nature of DXR between *P. falciparum* and *E. coli* and their similar mechanism of inhibition by FSM to study resistance mechanisms in *E. coli* as a proxy for *P. falciparum*.

In our study, we examined a published crystal structure of the *E. coli* DXR bound to FSM and selected the sites proximal to the FSM, DXP, and NADPH binding domains for saturation (Figure 4B). Thirty-three amino acids were selected for complete saturation to form an overall library of 660 mutants (amino acids were also silently mutated for control purposes). These mutations were generated directly at the *E. coli* genome level as previously reported [35]. Editing cassettes were synthesized using massively parallel DNA synthesis, and these cassettes were used as templates for recombineering using the lambda phage system [68,69]. Each editing cassette harbored two mutations: the first was the desired mutation while the second was a silent CRISPR protospacer-adjacent motif (PAM) mutation. Since the PAM is essential for the CRISPR system to fully recognize its target sequences, successfully edited cells will not be targeted, and their genome will not undergo a double-strand break—a lethal event in *E. coli* [70]. Following the construction of the genome-edited library, the cells were incubated in the presence of FSM to enrich for mutations that confer resistance, then were deep-sequenced to identify the mutations. Indeed, several mutations that induce FSM resistance were identified [40]. Importantly, thanks to the conserved nature of *dxr*, the identified sites also exist in other organisms, including *P. falciparum* and *P. vivax* strains (Figure 4C).

Among the resistant mutations, the mutation of a proline to a charged amino acid in position 274 was repeatedly identified. Indeed, the mutation of this proline to positively charged amino acids lysine and arginine resulted in increased half-maximal effective concentration (EC_50_) values compared to the wild type DXR (6.7, 5.5, and 1.2, respectively). The resistance mechanism of these mutations may be explained by the structural analysis performed by Yajima et al. where the proline residue and the FSM backbone sandwiched Trp212 in between, thus stabilizing the loop formation [71]. This structure is further stabilized by Met214 and His209. Interestingly, Met214, His209, and Trp212 were all targeted in the library, but none of them were enriched following FSM treatment. Other resistant mutations that were identified in positions 186 and 230 are less straightforward and will require further analysis to elucidate their resistance mechanism.

## 5. The Use of Surrogate Organisms

The approach of using *E. coli* as a platform for the discovery of drug-resistant mutations has several advantages and disadvantages. High-throughput genome editing methods have primarily been developed for laboratory strains such as *E. coli* and *S. cerevisiae*. While some methods are being adapted for an increasing number of non-model organisms and advances in *Plasmodium* genome editing have been reported [72,73,74], technologies for the high-throughput genome editing of *Plasmodium* strains will likely always lag after canonical model organisms. In addition, working with model organisms allows for experimentation in a standard molecular biology laboratory without extraordinary biohazard requirements. The distinct disadvantage of working on a different and distant organism is that there is no assurance that the same mutants will confer resistance in the actual organism of interest. Moreover, drug compatibility between species is not guaranteed, as in the case of MMV00813, which inhibits *Plasmodium* IspD, but has little effect on the *E. coli* ortholog [75]. We assume that *E. coli* can, in some cases, serve as a surrogate to narrow down the mutant candidates that will later need to be verified in the target organism. An alternative approach could involve using CRISPR-based tools such as those described by Bassalo et al. to integrate the *P. falciparum ispC* gene onto the *E. coli* genome in place of its native counterpart [76]. The *P. falciparum* and *E. coli* DXR genes are highly conserved; therefore, it is conceivable that the *Plasmodium* DXR may be functional in the context of an *E. coli* host. With the native *E. coli ispC* gene replaced with the *Plasmodium* sequence, saturation mutagenesis of critical residues in the active site of DXR may be performed and the library of mutants can be screened for FSM resistance in the context of a non-pathogenic model organism. However, it should be noted that in order to increase the probability of successful expression, sequence adaptations such as codon optimization (or harmonization), while maintaining the mRNA secondary structure may be required [77,78,79,80].

## 6. Discussion and Future Directions

High-throughput mutagenesis technologies are powerful tools for the prediction of mutations conferring drug resistance. However, these technologies still require further optimization, mainly in terms of editing efficiency [81]. For example, when using CRISPR systems, different guide RNA designs vary in performance, leading to a wide editing efficiency distribution across mutations [82,83]. Another consideration is that since the whole library is tested in a single culture, the dominant mutant is not only the one that grants resistance, but is also the one that has the least damage to its fitness. This additional factor might obscure important resistant mutants with lower growth rates [34,84], and may partially explain why, in our search for FSM-resistant mutations, we failed to isolate the previously reported S222T mutant, despite it being included in the library [41].

We propose that this technology can be further matured to be exploited at multi-gene and genome scales to deconvolute multi-mutational genotypes and pair inhibitors with their molecular targets. Chemical genetics approaches have been successfully applied to elucidate novel pairs of inhibitor molecules and their specific targets in *P. falciparum* [85,86,87,88,89,90,91,92,93]. Typically, these screens begin with applying inhibitors to the organism, followed by sequencing the genome of resistant strains to identify where mutations have arisen. Wherever possible, these studies are further validated with biochemical assays to substantiate the relationship between the inhibitor–target pair, as not all enriched mutations may result in the resistant phenotype. Demultiplexing all mutations found in resistant strains and individually inducing them will help to resolve the resistance-driving mutations from the mutational noise.

Common resistance mechanisms may additionally be targeted for the development of inhibitors. For example, when challenging *E. coli* with rifampicin, mutations in both its specific target, the RNA polymerase beta subunit (*rpoB*), and a gene implicated in multiple resistance mechanisms (*marR*) were found to confer resistance [35]. Thus, building more comprehensive genome-wide mutant libraries may help to pair broader categories of inhibitors to their specific targets and elucidate mechanisms of resistance.

Further systematic approaches may also potentially be used for pairing inhibitors to their targets. For example, if a drug is known to inhibit a specific essential pathway as with FSM [56], saturation of the complete pathway can be done to identify the target gene. Finally, libraries may also be genome-wide, spanning complete open reading frames, promoters, and other genetic elements. Complete agnostic approaches for genome-wide targeting have been performed at the gene resolution to identify genes essential for cancer cell growth and resistance, but a deeper, single-nucleotide resolution is still technologically challenging due to the library size [94,95,96]. Current barriers to accomplishing truly genome-wide libraries include transformation and editing efficiencies in the host organism, as mentioned above. The continuous improvement of these aspects may make such comprehensive libraries feasible in the future.

Mapping resistant mutations is an important first step in the search for the next generation of drugs. These data should then be integrated into structural predictions and drug–target docking simulations to elucidate the molecular resistance mechanism. Drug derivatives and similar molecules can then be tested in silico to narrow down potential candidates that would later be tested against the original mutational library. In this scenario, a successful candidate will result in significantly less resistant mutations than the original drug.

While we focus here on malaria, these approaches are not limited to *Plasmodium* strains. Molecules that were shown to be effective against malaria have been demonstrated to inhibit the growth of numerous other pathogens, including Trypanosomatids, *Toxoplasma gondii*, *Entamoeba histolytica, Perkinsus marinus,* and *Cryptosporidium parvum* [97,98,99,100], and the field as a whole may benefit from studying inhibitors that target multiple pathogens [101]. Moreover, the principles discussed here are also relevant for resistance phenotypes in general, from bacterial infections to the resistance of tumor cells in cancer patients.
